# Beyond CT accreditation: Systematic evaluation of achievable image quality, radiation dose, and protocol factors in annual physics testing

**DOI:** 10.1002/acm2.70366

**Published:** 2025-11-18

**Authors:** David W. Jordan, Christopher C. Smitherman, Jake M. Bell, William E. Moloney, Thomas J. Petrone

**Affiliations:** ^1^ University Hospitals Cleveland Medical Center, Department of Radiology Case Western Reserve University School of Medicine Cleveland Ohio USA; ^2^ Department of Imaging and Radiation Safety Hartford Healthcare Hartford Connecticut USA; ^3^ Alliance Medical Physics Alpharetta Georgia USA; ^4^ Bio‐Med Associates Inc. Flemington New Jersey USA; ^5^ Petrone Associates LLC Staten Island New York USA

**Keywords:** accreditation, ACR, CT, protocols

## Abstract

**Purpose:**

The purpose of this study was to assess the parameters and performance of CT protocols collected in numerous annual medical physics CT equipment performance evaluations (EPEs), develop clinically relevant performance targets for select phantom tests, and provide guidance for medical physicists about appropriate CT imaging parameters.

**Background:**

The results of CT scanner annual physics testing depend on the clinical protocols used at the facility, with a key image quality parameter dependent on the associated radiation dose. The American College of Radiology (ACR) accreditation process and quality control manual impose criteria on these parameters, but these minimum criteria are not meaningful performance targets for clinically acceptable protocols.

**Methods:**

A mathematical relationship between dose and contrast‐to‐noise ratio (CNR) was developed to evaluate test results and guide protocol adjustments. Historical testing results, including CNR and measured CTDI_vol_ were collected from 111 completed annual EPEs, and the results were compared to ACR criteria and published guidance on scanning parameters.

**Results:**

The average and 75th percentile CDTI_vol_ values were markedly lower than the ACR reference values, while the average and 25th percentile CNR values were markedly higher than the ACR minimum reference values. Scanners where iterative reconstruction (IR) was used did not show lower CTDI_vol_ values than scanners using filtered backprojection. Protocol parameters in routine clinical use showed high rates of deviation from published reference protocols and clinical imaging guidelines.

**Conclusions:**

Modern CT scanners likely can exceed ACR accreditation targets for image quality at radiation dose levels well below the ACR limits. Clinical medical physicists can use the approach described in this study to recommend clinical protocol improvements when performing physics testing. Medical physicists can engage with radiologists and technologists to determine whether published protocol guidelines are appropriate for clinical needs and can use the simple mathematical relationship described in this paper to translate protocols for modifications such as reduced slice thickness.

## 1 INTRODUCTION

In the United States and elsewhere, the testing instructions for the American College of Radiology (ACR) CT Accreditation Program (CTAP) and the ACR CT Quality Control Manual comprise the typical testing protocol for annual medical physics equipment performance evaluation (EPE) of CT scanners in diagnostic imaging. These procedures consist largely of scanning standardized phantoms for dose and image quality assessments using the facility's typical clinical imaging protocols for adult head, adult abdomen, pediatric head, and pediatric abdomen, rather than a standardized set of test scanning parameters. Accordingly, the results of physics testing are strongly influenced by the facility's patient imaging protocols in addition to the underlying technical performance of the CT scanner.

ACR CTAP applies minimum passing criteria for these phantom tests, and these criteria are typically used in medical physicist evaluation reports for CT equipment. In the authors’ clinical experience, one test in particular, the phantom contrast‐to‐noise ratio (CNR)—like the earlier test it replaced, visual assessment of low contrast detectability—depends strongly on the radiation output prescribed in the scanning protocol and seldom fails due to scanner performance; inadequate CNR (below the ACR minimum passing value) can usually be corrected by increasing the scanner radiation output setting for the clinical protocol. These adjustments can usually be made while keeping dose index values well within the ACR‐recommended reference ranges for each protocol. Since phantom test criteria reflect minimum performance, medical physics test results referenced to the ACR CTAP criteria do not meaningfully guide opportunities for improved clinical imaging performance; additional targets or reference levels are needed to guide improvements. This suggests a need for more meaningful performance targets for these tests.

Medical physicists are charged with periodically reviewing CT protocols in addition to testing equipment.^1^ Detailed information about protocol parameters is routinely collected for annual equipment testing purposes, and ACR and the American Association of Physicists in Medicine (AAPM) publish guidance that medical physicists can use in evaluating protocol parameters beyond verifying the corresponding dose levels and phantom test performance. In practice, there is substantial variation in CT protocol parameters used across geographic areas and variegated fleets of CT scanners, all of which pass ACR accreditation criteria. There is also inconsistency in the use of iterative reconstruction (IR) techniques and the associated radiation dose reductions they are supposed to enable, which have generally fallen short of the marketing claims made by CT equipment manufacturers.^2^, ^3^, ^4^, ^5^ This suggests an opportunity for medical physicists to make more robust recommendations about protocols when participating in protocol reviews and scanner testing.

The purpose of this study was to assess the parameters and performance of CT protocols collected in numerous annual medical physics CT EPEs, develop clinically relevant performance targets for select phantom tests, and provide guidance for medical physicists about appropriate CT imaging parameters. The specific aims were to determine clinically derived target values for phantom CNR and CTDI for the four commonly assessed clinical protocols, to assess conformance of clinical CT protocols to evidence‐based and consensus protocol recommendations, to evaluate the extent to which IR was used to reduce patient radiation dose, and to develop a figure of merit linking CNR and dose.

## 2 MATERIALS AND METHODS

### 2.1 Relationship between CNR and scan parameters

In the ACR CTAP, the CNR is measured by placing two circular regions of interest (ROI) on a specific phantom target and a nearby background region where the typical contrast between the target and background material is approximately 7 HU. The size and placement of the regions are illustrated in the ACR CTAP Phantom Testing Instructions^6^ and the ACR CT Quality Control Manual.^7^ The CNR is calculated as the difference (in HU) between the two ROI means divided by the ROI standard deviation for the background ROI. We denote this value as CNR_ACR_ to indicate that while it is calculated and reported per the ACR instructions, it is not a true CNR calculated from the standard error of the pixels of the measured object.

For a CT acquisition and reconstruction using specified values of kVp and reconstruction kernel, the noise depends in a predictable way on the reconstructed image thickness *T* and the radiation output of the scanner represented by CTDI_vol_. We introduce a proportionality constant *η* for the fixed parameters such that CNR_ACR_ varies with reconstructed slice thickness and radiation dose according to Equation (1):

(1) $$\mathrm{CN}R_{\mathrm{ACR}}=\eta \times \sqrt{\mathrm{CTD}I_{\mathrm{vol}}}\times \sqrt{T}$$

In Equation (1), CTDI_vol_ varies with the scanner's tube current‐rotation time product (mAs) but not with other parameters that normally influence contrast, CTDI_vol_, or both, such as kVp or collimation. The effects of those parameters are incorporated into the value of *η*.

*η* can be interpreted as a dose efficiency factor relating the image CNR_ACR_ to the CT scanner radiation output delivered to the imaged volume during the acquisition.

Using Equation (1), we determined the minimum value of *η* needed to satisfy the ACR criteria for passing phantom test results for each of the four clinical protocols. For each protocol, we set CNR_ACR_ equal to the minimum passing value for the protocol, *T* equal to the maximum slice thickness (mm) from the ACR CTAP clinical image guidelines, and CTDI_vol_ equal to the ACR‐recommended reference level (mGy). Solving for *η*, we find that each protocol would need to have *η* equal to the following values to produce minimally passing phantom test results:

*η*_min _= 0.05 for adult head;
*η*_min _= 0.089 for adult abdomen;
*η*_min _= 0.05 for pediatric head; and,
*η*_min _= 0.065 for pediatric abdomen (using 32 cm phantom with 7.5 mGy CTDI_vol_ reference)

### 2.2 Data from Annual Physicist Survey Reports

We collected 111 annual medical physicist CT EPE reports from the records of two medical physics service groups, detailing testing conducted over a period of 3 years in hospitals and outpatient imaging centers in New York and Ohio. A summary of collected data is provided in Table 1.

**TABLE 1:** Number of surveys from which data were collected for each clinical protocol type. Number of protocols collected:

	Adult head	Adult abdomen	Ped head	Ped abdomen
GE	42	43	40	41
Siemens	26	27	26	26
Philips	19	19	19	19
Toshiba	21	22	20	21
(Total all mfrs)	108	111	105	107
FBP	83	80	83	76
Iterative reconstruction	25	31	22	31

*Note*: Not all reports collected included data from all four protocols. Some scanners are used only for adult scanning and were not tested for pediatric imaging, and a few scanners were used only for body imaging and were not tested for head protocols.

Abbreviations: FBP, filtered back projection.

From each physics report, the following data elements were recorded for each of the four clinical protocols (adult head, adult abdomen, pediatric head, and pediatric abdomen) used for ACR phantom testing: scan mode (axial or helical), kVp, mA, rotation time (s), pitch, effective mAs, number of detector rows (N), detector row width (T, mm), reconstructed image thickness, reconstruction type (filtered back projection [FBP] or IR), reconstruction kernel, reconstruction options, displayed CTDI_vol_, measured CTDI_vol_, and measured phantom CNR_ACR_. For pediatric abdomen protocols, the CTDI_vol_ was measured using the 32 cm CTDI phantom. The scanning parameters are the same as those required to complete the Phantom Site Scanning Data Form for ACR CT accreditation. We also recorded the date of the survey, scanner manufacturer, scanner model, facility name or ID, and scanner ID or room number for each EPE.

CTDI_vol_ measurements were performed using standardized phantoms, instruments, and protocols, but the phantom and instrument serial numbers and physicist names were not collected. Each reported CTDI_vol_ value was the final result, including correction for appropriate calibration factors for the instrument used.

We calculated the median and 75th percentile CTDI_vol_ value for each of the four protocol types for comparison to the published U.S. achievable dose (AD) and diagnostic reference level (DRL) values, respectively, in patient exams from the ACR Dose Index Registry.^8^, ^9^ In pediatric head exams, the measured values are specific to patients aged 18 months per ACR CTAP guidelines, and we compared these values to benchmark values for the 1 to < 2 year age group. Pediatric abdomen data are for protocols used for patients weighing 40 to 50 pounds (18 to 23 kg); these are compared to benchmarks for the 5 to < 10 year age group following the weight‐to‐age conversion used by Kanal et al.^9^

The CNR_ACR_ measurements were performed according to the ACR testing instructions using Gammex model 464 ACR CTAP phantoms (Sun Nuclear, Middleton, Wisconsin, USA). The surveys were performed by multiple medical physicists using multiple phantoms; the phantom serial number and physicist name were documented in each report, but these details were not collected for this study.

Clinical images were not evaluated for quality in this study. The protocols on each scanner were tested as found during each annual EPE. We presumed that these protocols produced acceptable clinical image quality in routine imaging and represented the results of site‐specific adjustments made during initial setup of each scanner or based on subsequent radiologist feedback during routine use.

We assessed clinical protocol parameters for agreement with the reference CT protocol recommendations published by AAPM^10^, ^11^, ^12^, ^13^ and the CT Clinical Image Quality Guide published by ACR for its accreditation program.^14^, ^15^, ^16^, ^17^

We calculated the target *η* value for desired clinical performance from the collected physics survey data using the median CNR_ACR_, 75th percentile CTDI_vol_, and mode of slice thickness for each protocol type. Since these parameters represent clinical scanning performance that was acceptable to interpreting radiologists for each site, they represent an implied consensus of acceptable CNR_ACR_ and slice thickness. Using the 75th percentile CTDI_vol_ is consistent with the ACR CTAP methodology, which provides reference level recommendations for CTDI_vol_ for phantom testing. When the DRL concept is extended from dose indices to noise values, the median noise value is used as the reference level.^18^ We follow that approach in using median CNR_ACR_ to calculate the target *η*.

## 3 RESULTS

### 3.1 Measured CTDI_vol_

**FIGURE 1:**
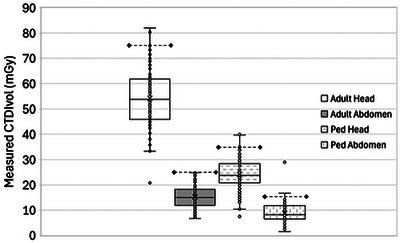
CTDI_vol_ measurements (in mGy) for four protocols in 111 annual CT EPE. The dashed horizontal line with diamond markers on each series indicates the ACR CTAP reference level value. ACR, American College of Radiology; CTAP, CT Accreditation Program; EPE, equipment performance evaluation.

The measured CTDI_vol_ values for the four protocols are plotted in Figure 1. For each protocol, the median and 75th percentile CTDI_vol_ values were markedly lower than the ACR reference value.

Table 2 compares these findings with ACR Dose Index Registry AD and DRL values.^8^, ^9^ The values for these sites and scanners were similar to, but slightly higher than, the U.S. registry values, with the exception of the DRL for adult abdomen, which was marginally lower in our study.

**TABLE 2:** Comparison of achievable dose and diagnostic reference level CTDI_vol_ values for this study and previously published U.S. data for adult and pediatric CT exams.

	This study	ACR registry data^8^, ^9^
**Adult head**		
Achievable dose (mGy)	53.8	49
Diagnostic reference level (mGy)	61.9	57
**Adult abdomen**		
Achievable dose (mGy)	15.0	13
Diagnostic reference level (mGy)	18.25	19
**Pediatric head**		
Achievable dose (mGy)	23.8	22
Diagnostic reference level (mGy)	28.4	27
**Pediatric abdomen**		
Achievable dose (mGy)	8.2	6.2
Diagnostic reference level (mGy)	11.8	8.1

Abbreviations: ACR, American College of Radiology.

### 3.2 Measured CNR_ACR_

**FIGURE 2:**
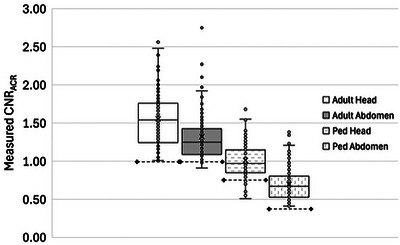
Phantom CNR_ACR_ measurements for four protocols in 111 annual CT EPE. The dashed horizontal line with diamond markers on each series indicates the ACR CTAP minimum required value. ACR, American College of Radiology; CNR, contrast‐to‐noise ratio; CTAP, CT Accreditation Program; EPE, equipment performance evaluation.

The results of the measured CNR_ACR_ values for the four test protocols are shown in Figure 2. For each protocol, the median and 25th percentile CNR_ACR_ were markedly higher than the ACR minimum reference value.

### 3.3 Relationship between CNR and scan parameters

We calculated target clinical *η* values using the median CNR_ACR_, 75th percentile CTDI_vol_, and typical slice thickness values determined from our field data collection. The results of these calculations for all four protocols are summarized and compared to the ACR minimum criteria in Table 3. This table shows suggested CNR_ACR_ and CTDI_vol_ targets for good clinical performance as well as the corresponding minimum and target *η* values that can be used to guide adjustments to a particular protocol.

**TABLE 3:** Comparison of ACR minimum performance and clinically realistic performance measured in the study data.

Protocol	Minimum *η*_ACR_	Study median CNR	Study 75% CTDIvol	Study typ. slice thk (mm)	Minimum *η*_target_
Adult head	0.05	1.54	61.9	5	0.0875
Adult abdomen	0.089	1.25	18.25	5	0.13
Pediatric head	0.05	0.97	28.4	5	0.08
Pediatric abdomen	0.065	0.67	11.8	3	0.11

Abbreviations: ACR, American College of Radiology; CNR, contrast‐to‐noise ratio.

**FIGURE 3:**
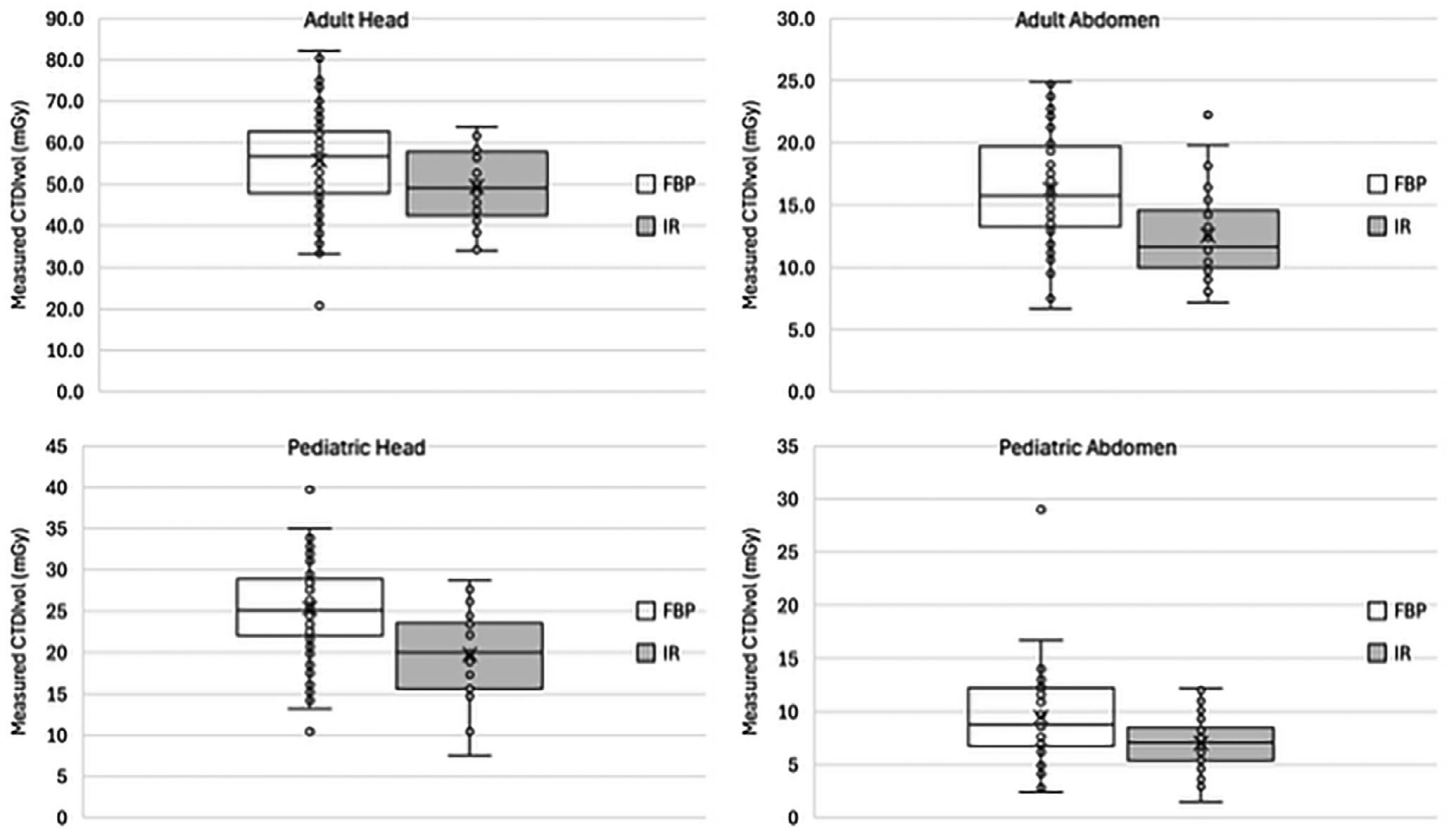
Comparison of measured CTDI_vol_ values for protocols using FBP reconstruction versus those using IR. FBP, filtered back projection; IR, iterative reconstruction.

### 3.4 Comparison of CTDI_vol_ for FBP and IR protocols

The results of comparing CTDI_vol_ values for scans using FBP and IR for the four clinical protocols are shown in Figure 3. The data show that scanners and protocols using IR did not use lower CTDI_vol_ than scanners and protocols using conventional FBP.

### 3.5 Protocol parameters

The adult head protocol reconstructed image thickness deviated from that recommended by the AAPM reference protocol in 26.5% of surveys. 45.6% of adult abdomen protocols deviated from the AAPM‐recommended image thickness. Among pediatric head protocols, 50.7% deviated from the AAPM‐recommended image thickness. AAPM reference protocols recommend different image thicknesses for different scanner models and patient sizes; in this study, the most common image thickness in use for pediatric abdomen was 3 mm, whereas ACR CTAP allows up to 5 mm.

Head protocols showed wide variation in the tube current (mA) value used. 92.3% of adult head protocols deviated from the AAPM‐recommended scanner‐specific mA value, and 58.9% of the protocols used higher mA values than recommended. For pediatric head protocols, 100% deviated from AAPM recommendations, with 40.8% using higher mA values than recommended.

Adult abdomen protocols showed variations in the helical pitch value used. 54.9% of the collected protocols deviated from the AAPM recommendation, and 40.8% of the protocols had lower pitch values than recommended.

Pediatric abdomen protocols showed variation in the kVp value used, which affects both contrast and patient dose. Pediatric body protocols’ kVp values deviated from the AAPM recommendations in 39% of the annual EPE reports.

## 4 DISCUSSION

The results of the measured CTDI_vol_ and CNR_ACR_ show that the ACR CTAP phantom criteria are not meaningful clinical performance targets for modern CT scanners, as the units sampled routinely achieved CNR_ACR_ performance above the minimum requirements at dose (CTDI_vol_) levels below the accreditation maximum limits. Introducing the figure of merit *η* provides some insights into the relationship between these parameters and clinical performance.

Table 3 shows that clinical scanners operate at dose efficiencies of about 150% to 200% of the minimum performance needed to pass ACR CTAP accreditation. One possible interpretation is that the scanners included in our study have opportunities for dose reduction; this assumes the ACR CTAP minimum CNR_ACR_ values correlate to adequate clinical image quality. Another is that the scanner capabilities allow latitude for radiologists and medical physicists to select the clinical operating point for each protocol. This opportunity will likely increase with the ongoing introduction of improved noise reduction reconstructions and technologies such as photon‐counting CT. Figures 1 and 2 demonstrate that there is wide variability in practice that could potentially be reduced by targeting benchmark image quality performance for the appropriate slice thickness and using the target *η* values in this paper to determine the required CTDI_vol_.

In this study, we observed that most scanners equipped with IR used it to some extent but did not meaningfully reduce CTDI_vol_ compared with scanners or protocols using FBP. Investigation of the reasons for this was beyond the scope of this study, but clinical experience suggests that adoption of IR varies due to inconsistency in site training and implementation support from vendors and hesitation by interpreting physicians to make changes affecting image texture. Regardless of the underlying cause, these findings reinforce the understanding that most commercial implementations of IR in CT have the potential to reduce dose by requiring less exposure to achieve similar noise levels, but that such reductions are often not directly linked to activation of the IR feature and must be adjusted separately.

The observed variation in acquisition and reconstruction parameters illustrates an opportunity for medical physicists to add value during EPEs by highlighting differences between site parameters and common guidelines such as the ACR accreditation program documents and the AAPM reference CT protocols. Sites may have valid reasons for deviating from these typical values, and if so, the medical physicist should discuss with the radiologist and technologist to understand those reasons. Where these deviations result from inattention to guidelines or defaulting to protocol presets provided by vendors or peers, there is an opportunity for the medical physicist to work with the facility to make improvements in parameters that the medical physicist must collect as part of the annual physics EPE anyway.

### 4.1 Limitations

A limitation of this study is the use of a simple ROI‐based CNR_ACR_ measurement, measuring noise amplitude and ignoring the effects of noise texture and object size, for IR protocols. Such measurements of CNR_ACR_ have been shown^19^, ^20^ to be inadequate for evaluating image quality performance for such images, and a more rigorous evaluation of IR CT images would use a task‐based performance evaluation such as the methods described in AAPM Report 233.^21^ In this study, we used the values measured exactly as described in the ACR CTAP instructions, not because they are fully adequate surrogates of clinical performance, but because this is the *de facto* standard for quality control measurements performed annually by clinical medical physicists and the basis for quantitative criteria used by the ACR CTAP to evaluate accreditation submissions. Scaling protocol parameters based on *η* values is likely to be inadequate for optimizing IR protocols, although these calculations may still be useful in assessing the performance of a scanner and protocol relative to the ACR accreditation criteria.

Another limitation related to IR is that while we collected data indicating whether IR was used for each scanner and protocol, we did not capture details of the type of method used or the user‐selectable settings. Commercial CT IR methods have a variety of noise reduction strengths and other changes to image appearance, and we did not investigate these aspects in detail in this study. We therefore cannot determine whether the similarity in CTDI_vol_ values observed between IR and non‐IR protocols was caused by sites failing to reduce CTDI_vol_ when using IR or whether the IR methods and settings used did not reduce noise sufficiently to enable larger CTDI_vol_ reductions.

### 4.2 Clinical implementation recommendations

The practicing clinical imaging physicist can use the results presented in this paper to streamline CT scanner performance testing and make meaningful improvements in clinical CT protocols. We present several approaches suggested by our findings.

#### 4.2.1 Assess scanner minimum performance capability

During physics testing, calculate *η* for each protocol and compare it to the minimum values for ACR CTAP passing criteria. If *η* exceeds the minimum, then it is possible to simply increase mA to correct insufficient CNR_ACR_ or decrease mAs to correct excessive dose while staying within the ACR maximum dose and minimum CNR limits.

#### 4.2.2 Apply clinical targets for image quality

If CNR_ACR_ values are less than target values in Table 3 for FBP reconstructions, consult with the site radiologist to assess clinical image quality. Similarly, if CTDI_vol_ is greater than the DRL values in Table 3, the medical physicist should investigate dose reduction opportunities with the radiologist and technologist. Calculate *η* for the as‐found protocol; if the value is greater than the Table 3 target value, then the protocol can be modified either to achieve the target CNR_ACR_ at a CTDI_vol_ less than the DRL or a CTDI_vol_ at the DRL yielding above‐target CNR_ACR_. If the *η* value is below the target, then other protocol changes are needed to reach the targets in Table 3. Check that kVp conforms to published guidelines and test other reconstruction kernels.

#### 4.2.3 Prospectively evaluate practice updates

While the ACR CTAP specifies a maximum slice thickness of 5 mm for the adult abdomen protocol, and this value was the mode in this study, the use of 3 mm slice thickness is increasingly common in practice;^22^ medical physicists who encounter protocols using 5 mm slices in testing can directly examine the effect of using 3 mm slices. At a clinical target *η* of 0.13, a CNR_ACR_ value of 1.25 in a 3 mm slice would require a CTDI_vol_ of 30.8 mGy. The minimum CNR_ACR_ value of 1.0 for a 3 mm slice width would require a CTDI_vol_ of 19.7 mGy for the same *η* of 0.13. Thus, 3 mm slices can be used at an increased dose to maintain CNR_ACR_ or at a reduced CNR_ACR_, or other reconstruction parameters can be tested to find higher *η* values.

#### 4.2.4 Track longitudinal scanner performance

Tracking *η* during sequential annual tests and comparing to a baseline value can alert the medical physicist to changes in the condition of the equipment and counter the tendency to compensate through dose creep. Because of the wide latitude between ACR minimum criteria and typical clinical results, it is not unreasonable to expect that a scanner experiencing a gradual increase in noise could be adjusted with small increases in radiation output to compensate, while staying within the reference values recommended by ACR. This would unnecessarily increase patient doses while overlooking the scanner degradation needing repair or calibration. Benchmarking and tracking *η* values would make these changes more readily apparent and facilitate earlier detection and correction.

#### 4.2.5 Compare performance between scanner models

The *η* value may provide a useful performance comparison between different scanner models or manufacturers. While *η* does not directly provide a means to match protocols or image characteristics among disparate scanners, it can be used to illustrate differences in capabilities among a variegated fleet of scanners. A practice could establish a typical range of *η* values for its fleet and use this range to decide when an older scanner, or one lacking certain features, can no longer deliver clinical performance on par with the rest of its fleet. Comparisons based on *η* can also be used to illustrate the dose cost or reduction that would be achievable when attempting to match image quality performance across a variety of scanners, or to predict how much variation in image quality could be expected if a group of scanners had their protocols matched based on dose index values.

## 5 CONCLUSIONS

This survey of a large data set from annual testing of clinical CT scanners shows that sites usually achieve CNR_ACR_ values above the ACR accreditation minimums with CTDI_vol_ values lower than the ACR reference values for the four commonly tested clinical protocols. Medical physicists can use the targets suggested in this study to recommend clinical protocol improvements when performing physics testing.

Our results showed that as‐found key parameters in clinical CT protocols, such as slice thickness, frequently deviated from published guidelines. Medical physicists, radiologists, and technologists should work together to determine whether these deviations represent improvement opportunities or whether they are justified by local practice needs.

The figure of merit *η* links the related parameters of CTDI_vol_ and image CNR_ACR_ to guide medical physicists in prospectively estimating the effect of changing one or the other of these parameters after making a single measurement of both values for a given scanner and protocol. This value can be interpreted as a dose efficiency factor for the scanner and protocol to deliver the target CNR_ACR_ in CT images.

## AUTHOR CONTRIBUTIONS

David W. Jordan and Thomas J. Petrone developed the concept for the study. All authors contributed to the collection and analysis of the data. David W. Jordan, Christopher C. Smitherman, and Jake M. Bell drafted the manuscript, and all authors contributed to the revision and editing of the manuscript and gave approval for the final submitted version.

## CONFLICT OF INTEREST STATEMENT

The authors have no relevant conflicts of interest to disclose.
